# Detection of circulating tumor DNA in colorectal cancer patients using a methylation‐specific droplet digital PCR multiplex

**DOI:** 10.1002/1878-0261.70161

**Published:** 2025-11-14

**Authors:** Luisa Matos do Canto, Louise Raunkilde, Jan Lindebjerg, Mads Malik Aagaard, Christina Therkildsen, Jakob Kleif, Lars Henrik Jensen, Torben Frøstrup Hansen, Rikke Fredslund Andersen

**Affiliations:** ^1^ Department of Biochemistry and Immunology Vejle Hospital, University Hospital of Southern Denmark Denmark; ^2^ Danish Colorectal Cancer Center South, Vejle Hospital University Hospital of Southern Denmark Denmark; ^3^ Department of Oncology Vejle Hospital, University Hospital of Southern Denmark Denmark; ^4^ Department of Clinical Pathology Vejle Hospital, University Hospital of Southern Denmark Denmark; ^5^ Department of Clinical Genetics Vejle Hospital, University Hospital of Southern Denmark Denmark; ^6^ Gastro Unit Copenhagen University Hospital, Amager ‐ Hvidovre Hospital Denmark; ^7^ Department of Clinical Medicine Faculty of Health and Medical Sciences, University of Copenhagen Denmark; ^8^ Department of Surgery Copenhagen University Hospital‐North Zealand Hilleroed Denmark; ^9^ Institute of Regional Health Research, Faculty of Health Sciences University of Southern Denmark Odense Denmark

**Keywords:** circulating tumor DNA, Colorectal Cancer, ddPCR, methylation

## Abstract

Despite the use of conventional biomarkers and imaging methods for treatment monitoring of colorectal cancer (CRC) patients, limitations remain in detecting minimal residual disease and early relapse. Circulating tumor DNA (ctDNA) offers a promising noninvasive and cost‐effective alternative for monitoring disease progression and relapse, potentially improving patient outcomes. In this study, we developed a methylation‐specific droplet digital PCR (MS‐ddPCR) multiplex assay designed to detect ctDNA through a combination of tumor‐specific and tissue‐conserved methylation markers. Our objective was to evaluate the performance of this assay in patients with CRC and assess ctDNA dynamics as a prognostic tool in those with metastatic CRC (mCRC). The assay demonstrated high specificity (96.7%) and sensitivity in detecting ctDNA in both patients with localized tumors (64.4%) and mCRC (89.2%). Notably, ctDNA dynamics from baseline to after the first treatment cycle were significantly associated with progression‐free survival (PFS) and overall survival (OS) in mCRC. Classifying patients based on ctDNA‐RECIST (Response Evaluation Criteria in Solid Tumors) and the percent reduction in ctDNA fraction revealed pronounced differences in PFS and OS. Median PFS and OS were 11.4 and 35.3 months for good responders compared with 7.6 (HR = 1.71, 95% CI 0.9–3.25) and 18.4 (HR = 2.15, 95% CI 1.16–3.99) for poor responders, while patients with progressive disease had a median PFS and OS of 5.1 (HR = 4.36, 95% CI 1.91–9.92) and 6.85 (HR = 4.73, 95% CI 2.09–10.7) months. Our multiplex MS‐ddPCR assay provides a sensitive, cost‐effective approach for detecting and quantifying ctDNA in CRC patients, especially in metastatic disease. The ability to monitor ctDNA dynamics holds potential for early treatment response assessment, prognosis, and guiding personalized therapeutic strategies, making it a valuable tool for clinical practice.

## Graphical abstract

We developed a cost‐effective methylation‐specific droplet digital PCR multiplex assay containing tissue‐conserved and tumor‐specific methylation markers. The assay can detect circulating tumor DNA with high accuracy in patients with localized and metastatic colorectal cancer. This tumor‐agnostic approach enables monitoring of treatment response and disease progression, supporting clinicians in early therapeutic decisions and follow‐up.

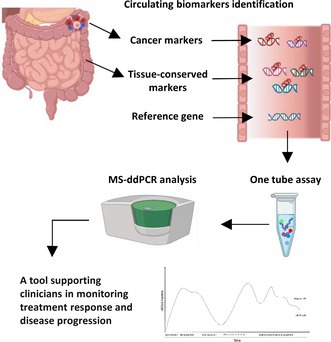

**Abbreviations**
AUC: area under the ROC curve
BSC: bisulfite‐converted
CI: confidence interval
CRC: colorectal cancer
ctDNA: circulating tumor DNA
DCCCS: Danish Colorectal Cancer Center South
ddPCR: droplet digital polymerase chain reaction
DMC: differentially methylated CpGs
GDC: Genomic Data Commons
GEO: Gene Expression Omnibus
HMW: high molecular weight
HR: hazard ratios
LoB: limit of blank
mCRC: metastatic CRC
MRD: minimal residual disease
MS‐ddPCR: methylation‐specific ddPCR
NCBI: National Center for Biotechnology Information
NCR: near complete response
NGS: next‐generation sequencing
NR: not reached
OS: overall survival
PD: progressive disease
PFS: progression‐free survival
PR: partial response
QC: quality control
RECIST: Response Evaluation Criteria in Solid Tumors
RFE: recursive feature elimination
ROC: receiver operating characteristic
SD: stable disease
TCGA: The Cancer Genome Atlas

## 1 Introduction

Colorectal cancer (CRC) is the third most incident type of cancer and the second leading cause of cancer‐related mortality in men and women worldwide [1]. Approximately 15–30% of patients have metastases at diagnosis, and 20–50% with localized disease will later develop them, most commonly in the liver, lungs, peritoneum, and distant lymph nodes [2]. With the rise of personalized medicine, matching the right treatment option with the right patient is challenging. Treatment monitoring and early detection of relapse are key to improving patient outcomes. A few biochemical markers (e.g., carcinoembryonic antigen) and imaging‐based methods, including RECIST (Response Evaluation Criteria in Solid Tumors) [3], are the standard for assessing treatment response but have limitations in detecting minimal residual disease (MRD) and early progression [4].

Liquid biopsy‐based biomarkers, such as circulating tumor DNA (ctDNA), have emerged as a noninvasive tool for cancer detection, disease monitoring, and prognostication. ctDNA is short DNA fragments shed or secreted into the bloodstream or other bodily fluids by cancer cells [5]. Because these cells undergo genetic and epigenetic alterations during tumorigenesis, it is possible to identify the DNA fragments by targeting cancer‐specific changes. While mutations in DNA sequence are specific to an individual tumor and quite heterogeneous in a patient population [6], DNA methylation modifications often occur early in carcinogenesis and can serve as universal biomarkers across tumors [7, 8, 9]. Furthermore, DNA methylation signatures are found to be specific for each tissue type, which provides another source for identifying the diseased organs that release DNA fragments into circulation [10, 11, 12]. Combining these two approaches may be employed to improve the sensitivity of ctDNA detection.

In CRC, ctDNA provides clinically relevant insights into early detection, prognosis, and treatment monitoring [7, 8, 13, 14, 15], with higher concentrations observed in patients with advanced disease [16]. Its detection is especially beneficial for assessing MRD, as persistent ctDNA after surgery or therapy is associated with an increased risk of recurrence [4, 17, 18, 19]. ctDNA levels fluctuate dynamically in response to tumor burden, treatment, and disease progression. Recent advancements have led to ctDNA‐RECIST, an adaptation of RECIST that evaluates treatment response based on ctDNA kinetics rather than tumor size changes on imaging [14]. This approach leverages the dynamic nature of ctDNA, where decreasing levels often precede radiological tumor shrinkage, while ctDNA rebounds may signal early progression or therapy resistance [9, 20].

Detection of ctDNA in plasma samples is commonly performed using next‐generation sequencing (NGS) or PCR‐based techniques, either separately or in conjunction, depending on the clinical application. While NGS provides comprehensive mutation profiling, droplet digital PCR (ddPCR) offers a highly sensitive, cost‐effective alternative with a shorter turnaround time, making it well‐suited for targeted biomarker detection. We and others have previously reported methylated *NPY* (Neuropeptide Y gene) as a universal CRC ctDNA marker using ddPCR and our results reinforce its value in early treatment response assessment and as a prognostic marker [8, 9, 21, 22, 23, 24, 25]. With advancements in ddPCR technology, analyzing several markers in a single reaction assay (multiplexing) has become increasingly feasible, enhancing sensitivity while reducing costs.

In this study, we developed a single‐tube multiplex methylation‐specific ddPCR (MS‐ddPCR) assay targeting a reference gene and five CRC‐related ctDNA methylation markers: two CRC‐specific markers and three novel colorectal tissue‐conserved markers. Our primary objectives were to evaluate the performance of the MS‐ddPCR assay in detecting and quantifying ctDNA in CRC patients and to determine whether changes in ctDNA levels correlate with disease progression and survival in metastatic CRC (mCRC).

## 2 Methods

### 2.1 In‐house samples

We used four distinct sets of samples in this study: (1) plasma from early‐stage CRC patients was used to determine the MS‐ddPCR assay cutoff and sensitivity; (2) serial plasma samples from mCRC patients included in a clinical trial, used to evaluate biomarker dynamics and survival outcomes; (3) plasma samples from healthy, FIT‐negative individuals participating in a CRC screening program, used to establish the assay's limit of blank (LoB) and specificity; and (4) normal and tumor tissue samples from a previously described cohort, used for biomarker validation and selection.

Retrospective analyses were performed on prospectively collected plasma samples from CRC patients at the Danish Colorectal Cancer Center South (DCCCS), Vejle Hospital, Denmark.

(1) Plasma samples from 59 patients diagnosed with colon or rectal cancer stage I‐III collected between October 2016 and June 2023, included in the Colorectal Cancer Database (CRC database) established at DCCCS, were used to identify the MS‐ddPCR multiplex assay cutoff and sensitivity. The Regional Committee on Health Research Ethics for Southern Denmark has approved the investigation (S‐20250011).

(2) Serial plasma samples collected between May 2016 and December 2018 from 65 CRC patients included in the phase II study registered at clinicaltrials.gov (Identifier NCT02705300, 10/03/2016) and previously described by our group [8, 26] were included for biomarkers dynamics and survival analyses. Patients presented with unresectable mCRC and were eligible for first‐line treatment with the FOLFOXIRI regimen (a combination of leucovorin, 5‐fluorouracil, oxaliplatin, and irinotecan). The inclusion criteria were colon or rectum adenocarcinoma, age 18–75 years, ECOG performance status (PS) 0–1 (patients above 70 years were eligible if PS = 0), and evaluable but not necessarily measurable disease according to RECIST 1.1. Previous adjuvant chemotherapy for radical treatment of stage II or III colorectal cancer was allowed in patients proven disease‐free for more than 6 months. The study was approved by The Regional Committee on Health Research Ethics for Southern Denmark (S‐20150185). Samples collected at baseline (before treatment) and after the first treatment cycle (after 2 weeks, just before starting the second cycle) were used in the analysis. The data cutoff for updated survival analyses was performed on 1 November 2021. The median follow‐up time was 48.5 months [8].

(3) Samples collected between June 2020 and September 2021 at Hvidovre Hospital, Denmark, under the clinical project entitled *Endoscopy III* were used as a control cohort for establishing the assays' limit of blank (LoB) and MS‐ddPCR multiplex specificity (*N* = 60). Eligible study individuals were participants of the fecal immunochemical test (FIT)‐based screening for CRC. All individuals were FIT‐negative, with an equal distribution regarding age (50–79 years), sex, alcohol, and tobacco intake as well as differences in body mass index. All individuals had no cancer registered after 2 years from the blood draw. The study was approved by the Scientific and Ethical Committee (H‐4‐2013‐050) and the Danish Data Protection Agency (2007‐58‐0015/HVH‐2013‐022).

The characteristics of the individuals, whose plasma samples were used in the analyses, are described in Table 1.

**Table 1:** Characteristics of the individuals included in the study. mCRC, metastatic colorectal cancer.

Characteristics	Healthy controls *N* = 60	Localized CRC *N* = 59	mCRC *N* = 65
Age, median (range)	65 (54–79)	73 (41–96)	62 (41–75)
Gender F/M	30/30	30/29	39/26
Primary tumor location			
Colon		29 (49%)	47 (72%)
Rectum		30 (51%)	18 (28%)
Location metastatic sites			
Liver only			16 (25%)
Liver and other site(s)			34 (52%)
Nonliver site			15 (23%)
Number metastatic sites			
1 site			26 (40%)
> 1 site			39 (60%)
Unresected primary tumor			
Yes			44 (68%)
No		59 (100%)	21 (32%)

(4) Adjacent normal and cancerous colon and rectum tissue samples (10 samples from each type) from an independent previously described cohort, collected between February 2004 and July 2005 at the Department of Surgery, Vejle Hospital, Denmark, were stored at −20 °C in RNAlater™ (Ambion, Austin, TX, USA). They were used to evaluate individual biomarker levels in the different tissue groups and to select the candidates for plasma testing [27].

The investigation was conducted in accordance with the Declaration of Helsinki, Danish law, and European data protection regulations. Samples were collected with the understanding and written consent of each subject.

### 2.2 Target selection for multiplex design

#### 2.2.1 Discovery of colorectal tissue‐conserved biomarkers

An approach previously described by Raunkilde *et al*. [24] was used to identify methylation sites consistent in colorectal tissue regardless of tumor status and absent in other tissue types (tissue‐conserved markers). This analysis was exploratory and aimed to identify candidate sites to be tested in‐house using ddPCR. Briefly, publicly available data from tissue samples analyzed using the Infinium® Human Methylation450 BeadChip array (Illumina) were acquired: (1) adjacent normal tissue from colon and other organs and primary colon tumor tissue data from The Cancer Genome Atlas (TCGA) project were obtained from the GDC data portal (April 2020) [28], (2) previously published and publicly available datasets containing data from noncancerous samples were downloaded from NCBI Gene Expression Omnibus (GEO) [29], (3) an in‐house dataset consisting of 34 noncancerous and 23 cancerous colon tissue samples. In total, data from 919 noncolon normal samples (REST) were compared with 398 samples from the colon (COLON), including normal and cancerous, and all datasets are described in the Table S1.

After data acquisition, methylation probes with missing values were filtered out, followed by calculating mean beta‐values for all colon samples (COLON) and all noncolon samples (REST). Differentially methylated CpGs (DMC) were identified, and colon hypermethylated sites (with mean COLON beta > 0.75 and mean REST beta < 0.25) or colon hypomethylated sites (mean COLON beta < 0.25 and mean REST beta > 0.75) were selected for further analysis. Recursive feature elimination (RFE) using 10‐fold cross‐validation, repeated five times, was used to identify the DMCs that separate colon from noncolon samples. In parallel, Random Forest (10‐fold cross‐validation, repeated 10 times) was used to detect the most important DMCs that separated the samples in both groups. Finally, we merged the resulting top 10 DMC as the union of the two (RFE and random forest) analyses and performed a manual visual inspection of the beta value distribution using boxplots for each sample group.

All data analyses were performed in R (v. 4.0.5) using the packages caret (v.6.0‐92), randomForest (v. 4.6‐14), mlbench (v. 2.1‐3), pheatmap (v. 1.0.12), ggplot2 (v.3.3.5), dplyr (v. 1.0.7), readr (v.2.1.2), and minfi [30] in addition to functions in base packages.

#### 2.2.2 Colorectal cancer biomarkers selection

CRC biomarkers selected from the literature were also tested to be included in the MS‐ddPCR multiplex assay. Previous studies from our group [8, 9] and others [22, 25] have shown the potential of methylated CpGs in the *NPY* gene as a ctDNA biomarker in CRC plasma samples. Methylated regions in three other candidates (*CLIP4*, CAP‐Gly domain containing linker protein family member 4; *C9orf50*, Chromosome 9 open reading frame 50; and *KCNQ5*, potassium voltage‐gated channel subfamily Q member 5) were reported to have high sensitivity and specificity for blood‐based detection of CRC and were also tested [7].

### 2.3 Assay design

Primers and probes were designed to target methylation‐specific regions using the PrimerQuest Tool (https://www.idtdna.com/SciTools), and PCR products were checked using BiSearch: ePCR tool [31]. Candidate assays were initially tested in duplexes with a reference gene (albumin—*ALB*) [22], using probes labeled with FAM and VIC, respectively. Tests were performed on commercially available Universal Methylated and/or Unmethylated Human DNA Standard (cat. no.: D5014, Zymo Research, Irvine, CA, USA) controls, cancerous and noncancerous tissue samples (from colon and rectum), genomic DNA from whole blood, and plasma from healthy individuals. Assay selection criteria included ddPCR efficiency, unspecific signal in genomic DNA from whole blood, plasma from healthy individuals, or tissue. After selecting five candidates, the primers and probes' interaction was checked using the Thermo Fisher Multiple Primer Analyzer (available at https://www.thermofisher.com).

### 2.4 DNA isolation from tissue, whole blood, and plasma samples

Tissues were dissociated in a Dispomix Drive Dissociator (Scientific Equipment Repair, Palo Alto, CA, USA), and DNA was isolated from samples using the DNeasy blood and tissue kit (cat. no.: 69504; Qiagen, Hilden, Germany) according to the manufacturer's instructions, and eluted in 200 μL AE buffer. The QiaSymphony DSP DNA Mini Kit (cat. no.: 937236; Qiagen) on the QIAsymphony SP instrument was used to isolate genomic DNA from 200 μL whole blood samples. Extraction was performed according to the manufacturer's instructions, and DNA was eluted in 400 μL elution buffer.

Blood samples from controls and CRC patients were collected in EDTA tubes and processed within 2 and 4 h, respectively. Samples from controls were centrifuged twice at 3000 ***g*** for 10 min, while those from CRC patients underwent centrifugation at 2000 ***g*** for 10 min, and the plasma was stored at −80 °C until use. Before DNA isolation, a second centrifugation step at 10 000 ***g*** for 10 min was performed, and ~36 000 copies of the exogenous control cysteine‐rich polycomb‐like protein 1 (CPP1) were added to each plasma sample [32]. cfDNA was isolated from 4 mL plasma according to the manufacturer's instructions using the QIAsymphony DSP circulating DNA Kit (cat. no.: 937556) on the QIAsymphony SP instrument (Qiagen) and eluted in 60 μL QIAsymphony Plasma Elution Buffer and 140 μL H_2_O.

### 2.5 Quality control of plasma samples

Four quality control (QC) assays evaluating preanalytical processing were analyzed in multiplex using single ddPCR per sample as described previously [33]. Briefly, purification efficiency was measured as the recovered fraction of CPP1 after DNA isolation [32]. An assay targeting the VDJ rearranged IGH locus specific for B cells (PBC) was used to estimate lymphocyte DNA contamination, considered when PBC/cfDNA ratio > 2% [32]. Total cfDNA concentration and contamination with high molecular weight (HMW) DNA were assessed by quantifying a short (65 bp) and a long (250 bp) fragment of the *EMC7* (ER membrane protein complex subunit 7) gene [34].

### 2.6 DNA bisulfite conversion and multiplex analysis of methylated ctDNA

For methylation analysis, cfDNA was concentrated to 20 μL in Amicon Ultra 30 K 0.5 mL columns (cat. no.: UFC5030BK; Merck, Darmstadt, Germany) and bisulfite‐converted according to the manufacturer's protocol in a 150 μL reaction with the EZ DNA Methylation‐Lightning kit (cat. no.: D5031; Zymo Research) and eluted in 15 μL elution buffer. Positive (cat. no.: D5014; Universal Methylated Human DNA Standard, Zymo Research) and negative controls (genomic DNA from whole blood and water) were converted alongside the samples.

Bisulfite‐converted (BSC) DNA was analyzed in 20 μL methylation‐specific ddPCR containing 2× ddPCR Supermix for probes (no dUTP) (cat. no.: 186‐3025; Bio‐Rad, Hercules, CA, USA), probes, and primers (Table S2) for the five target biomarkers and *ALB*, and 5 μL template DNA. Positive and negative controls were included in each plate. Droplets were generated using an Automated Droplet Generator (Bio‐Rad). Duplicate ddPCRs per sample were performed on a Veriti™ Thermal Cycler (Applied Biosystems, Waltham, MA, USA) with the following PCR conditions: 95 °C for 10 min, 44 cycles of 95 °C for 15 s and 56 °C for 1 min, and 98 °C for 10 min using a 2 °C/s ramp rate. The QX600 Droplet Reader was used to generate the data, which was analyzed in the QXManager Software 2.2 (Bio‐Rad).

The linearity of the multiplex assay and the five respective single targets in duplex with *ALB* was tested using a 6‐point twofold dilution series of the Universal Methylated Human DNA Standard (cat. no.: D5014; Zymo Research) in a constant background of 5600 copies of BSC genomic DNA.

### 2.7 Data analysis

Fluorescence intensity for each of the six targets in the multiplex from a positive control in each plate was used to determine positive and negative clusters. The ratio between each target and *ALB* was used to compare tissue and whole blood samples. The number of positive droplets from each biomarker in plasma samples from controls and CRC patients with localized disease was used to estimate sensitivity, specificity, and AUC (area under the receiver operating characteristic, ROC, curve). The best threshold value for each biomarker was determined using Youden's index [35], and a combined cutoff including all five biomarkers was used to classify samples as ctDNA‐positive. This cutoff was based on the number of markers above the threshold, giving a specificity of ≥ 95%. ctDNA dynamics were evaluated in samples from mCRC patients based on quantitative changes in each marker's ratio and 95% confidence interval (CI), as previously described [20]. ctDNA‐RECIST criteria, as described previously [14] and summarized in Table S3 were applied, and further analyses were performed to classify changes into categorical variables (Table S4) and evaluate survival association. Survival analyses were conducted from the date of the first treatment until disease progression or death from any cause, or they were censored at the time of surgery or the last hospital contact (progression‐free survival: PFS). Overall survival (OS) was measured from the same starting point to the date of death. Follow‐up data were censored as of November 2021.

### 2.8 Statistical analysis

The Shapiro–Wilk normality test was used to evaluate the variables' distribution. Categorical variables were compared using Pearson's chi‐squared test or Fisher's exact test (*N* < 5) when appropriate. Mann–Whitney *U* and Kruskal–Wallis tests were used for group comparisons. Pearson's and Spearman's Rank correlation coefficients were used to test the correlation between continuous variables with normal and non‐normal distributions, respectively. Survival analysis was performed using the Kaplan–Meier estimator and compared by the log‐rank test. Cox regression models were used to estimate hazard ratios (HR). All statistical analyses were performed using R (v.4.3.2). Graphs were generated using the packages ggplot2 (v.3.5.1) and survminer (v.0.5.0). All tests were two‐sided; *P*‐values < 0.05 were considered statistically significant. Missing data were treated as missing and no imputations were performed.

## 3 Results

### 3.1 Assays optimization and selection

The *in silico* analysis resulted in a list of eight unique DMCs, tested as candidates for the multiplex assay along with the four previously described CRC markers (*NPY, CLIP4*, *C9orf50*, and *KCNQ5*). After selecting the assays based on the criteria described in the methods section, different multiplexes were tested. The assay described to target the *NPY* gene interfered with other assays in the multiplex. Therefore, another assay was designed in the same region, but on the antisense DNA strand (Table S2). The new *NPY* assay was submitted to the same selection criteria as the other assays. Because the original *NPY* assay has been previously evaluated on the same mCRC samples reported here [8], the results from both assays targeting *NPY* were directly compared, showing a strong correlation (Fig. S1A–C).

After selection and optimization, five markers were combined to compose the MS‐ddPCR multiplex assay. Three tissue‐conserved (*FRMD4B*, FERM domain containing 4B; *GDAP1L1*, ganglioside induced differentiation associated protein 1 like 1; *CELF2*, CUGBP Elav‐like family member 2) and two cancer‐specific (*NPY* and *CLIP4*) markers were combined with the reference gene (*ALB*) using six different fluorophores (Table S2) and their respective quenchers. Each marker cluster position and background level were visually inspected to ensure no primer‐probe cross‐reactions (Fig. S2A,B).

### 3.2 Assays linearity and sensitivity

The five selected assays displayed a very high positive linear correlation between expected values from a 6‐point twofold dilution series of Universal Methylated Human DNA Standard and the measured copies per reaction using individual assays in duplex with *ALB* or the 6‐target multiplex (Fig. S3A–E).

**Fig. 1:**
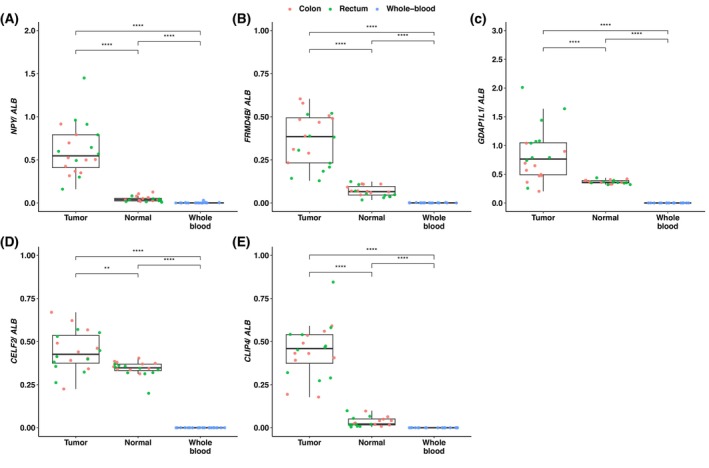
Methylation levels of the five targets included in the multiplex assay detected in colon and rectum tissue from normal (*N* = 20) and tumor (*N* = 20) samples, along with whole blood samples from healthy individuals (*N* = 20). Box plots show the ratio (median and interquartile range) of detected copies/μL of each marker and the reference gene (*ALB*). Mann–Whitney tests were used to define the statistical significance between each group (**: *P* ≤ 0.01; ****: *P* ≤ 0.0001).

All markers presented higher methylation levels in tumor tissue than in normal tissue and whole blood (Fig. 1A–E), with higher levels of tissue‐conserved markers (*FRMD4B*, *GDAP1L1*, and *CELF2*) in normal tissue (Fig. S4).

### 3.3 Quality control analyses of plasma samples

Recovery of total cfDNA yielded a median of 2600 copies/mL (range: 1046–16 520 copies/mL) in the control group, 2760 copies/mL (range: 1032–18 120 copies/mL) in patients with localized disease, and 4760 copies/mL (range: 1270–132 100 copies/mL) in baseline samples from CRC patients with metastatic disease. All analyzed samples passed QC. One sample presented an *EMC7* long/short ratio of 0.503, while all others had a ratio below 0.347. The percentage of CPP1 recovery was > 42%.

### 3.4 Levels of methylated markers in plasma samples from CRC patients

Clinical and histopathological characteristics of CRC patients were compared according to the percentage of methylated (target/ALB*100) markers detected in plasma samples at baseline. There was no significant association with gender, age, or primary tumor location in CRC patients diagnosed with localized disease (Fig. S5A–C). When evaluating clinical (c) and pathological stages (p), only *NPY* (median % methylated, IQR: cI = 0.152, 0.122; cII = 0.195, 0.163; cIII = 0.355, 0.503; *P* = 0.026) and *FRMD4B* (median % methylated, IQR: cI = 0.024, 0.038; cII = 0.123, 0.273; cIII = 0.142, 0.246; *P* = 0.018) showed a significantly higher percentage in patients with more advanced clinical stages (Fig. S5D,E). Furthermore, *NPY*, *FRMD4B*, and *GDAP1L1* presented significantly higher levels in plasma samples from patients with cT3‐4 than cT1‐cT2 tumors, while *NPY* and *FRMD4B* showed higher levels in cN+ cases. There were no significant differences in the methylation levels and pT or pN stages (Table 2).

**Table 2:** Median percentage of methylated targets detected by a methylation‐specific droplet digital PCR multiplex in plasma from colorectal cancer patients according to clinical and pathological stages. All patients were cM0 and pM0. IQR, interquartile range.

Characteristics	Median % methylated target (IQR) in patients with localized disease										
	Number of cases	*NPY*	*P*‐value	*FRMD4B*	*P*‐value	*GDAP1L1*	*P*‐value	*CELF2*	*P*‐value	*CLIP4*	*P*‐value
	59	0.302 (0.369)		0.122 (0.243)		0.141 (0.256)		0.087 (0.120)		0.047 (0.160)	
Clinical TNM											
cT1‐2	21	0.170 (0.192)	**0.018**	0.042 (0.105)	**0.018**	0.076 (0.128)	**0.018**	0.064 (0.045)	0.111	0.00 (0.064)	0.061
cT3‐4	38	0.352 (0.545)		0.192 (0.266)		0.192 (0.278)		0.120 (0.198)		0.064 (0.260)	
cN−	20	0.152 (0.157)	**0.009**	0.041 (0.188)	**0.021**	0.090 (0.124)	0.052	0.082 (0.100)	0.625	0.00 (0.078)	0.101
cN+	39	0.355 (0.503)		0.145 (0.246)		0.188 (0.292)		0.088 (0.175)		0.062 (0.230)	
Pathological TNM											
pT1‐2	8	0.161 (0.107)	0.058	0.039 (0.106)	0.146	0.080 (0.169)	0.111	0.061 (0.050)	0.194	0.011 (0.086)	0.429
pT3‐4	51	0.317 (0.406)		0.135 (0.259)		0.157 (0.287)		0.101 (0.121)		0.050 (0.176)	
pN−	29	0.313 (0.366)	0.295	0.145 (0.194)	0.399	0.144 (0.212)	0.649	0.112 (0.122)	0.48	0.047 (0.135)	0.881
pN+	30	0.246 (0.312)		0.084 (0.271)		0.130 (0.271)		0.079 (0.107)		0.039 (0.182)	

Bold values represent *P* < 0.05.

In patients with metastatic disease, no association with gender, age, primary tumor location, the number of metastatic sites, or whether the primary tumor was resected, was observed according to the percentage methylated levels of the five markers included in the multiplex assay (Fig. S6A–E).

### 3.5 Limit of blank and sample classification

**Fig. 2:**
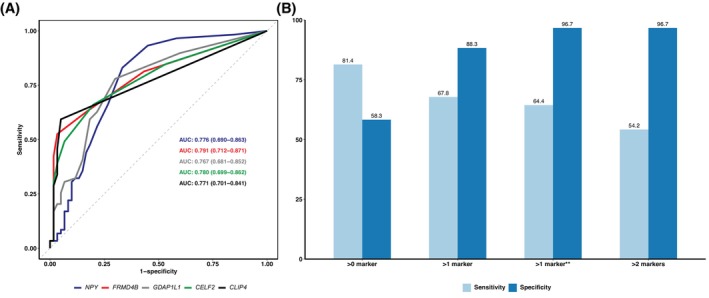
Potential of colorectal cancer (CRC) ctDNA markers, alone or in combination, to distinguish plasma samples from healthy donors (*N* = 60) and CRC patients with localized disease (*N* = 59). (A) Area under the receiver operating characteristics (ROC) curve (AUC) and 95% confidence intervals for each marker in the multiplex. The limit of blank (LoB) was established using Youden's index for each ROC curve. (B) The sensitivity and specificity of the combined markers, based on the number of markers above the LoB required to classify a sample as positive. ** A sample is considered positive when more than one marker is detected above the LoB, except when *NPY* and *GDAP1L1* were the sole positive markers.

The number of positive droplets detected in the plasma of 60 healthy controls and 59 CRC patients with localized disease was used to identify the LoB for each marker. The cutoffs were based on ROC analysis of the individual targets in the multiplex (Fig. 2A) and determined by the value giving the highest sensitivity–specificity combination (Youden's index). The LoB for each marker was *NPY* = 4, *FRMD4B* = 3, *GDAP1L1* = 2, *CELF2* = 2, *CLIP4* = 1. However, to classify a sample as ctDNA‐positive, a cutoff for the number of positive markers giving at least 95% specificity was investigated. Increasing the required number of markers above the LoB to call a sample positive leads to higher specificity with lower sensitivity (Fig. 2B), with more than two markers reaching > 95% specificity. In all controls with two markers above the LoB, we observed that the same pair of markers was present (*NPY* and *GDAP1L1*), while the same scenario was not seen in patient samples. Therefore, considering a sample ctDNA‐positive when more than one marker was above the LoB, except when *NPY* and *GDAP1L1* were the only positive markers, resulted in the best sensitivity–specificity combination (Fig. 2B). Using this classification, only two plasma samples from controls were positive (3.3%), 38 from CRC patients with localized disease (64.4%), and 58 from mCRC patients (89.2%) collected at baseline. Sensitivity, specificity, accuracy, positive, and negative predictive values of each marker and the MS‐ddPCR for identifying CRC patients with localized or metastatic disease are described in Table S5.

### 3.6 ctDNA positivity and clinical characteristics

There was no statistically significant association between preoperative ctDNA status in CRC patients with localized disease and clinical or histopathological characteristics. The percentage of positive cases according to clinical and pathological stages was: cI = 50%, cII = 60%, and cIII = 69.2%; pII = 62.1% and pIII = 66.7% (*P* > 0.05). A higher fraction of patients with a cT3–4 were ctDNA‐positive (74%) compared with cT1–2 (48%), with marginal significance (*P* = 0.045). No significant association between ctDNA status at baseline and patient characteristics (age, gender distribution, primary tumor location, number of metastatic sites, whether the primary tumor was resected) was observed in the group of patients with mCRC (Table 3).

**Table 3:** Patient characteristics and ctDNA status at baseline.

Characteristics	Localized disease		*P*‐value	Metastatic disease^a^		*P*‐value
	ctDNA‐negative (*n* = 21)	ctDNA‐positive (*n* = 38)		ctDNA‐negative (*n* = 7)	ctDNA‐positive (*n* = 58)	
Age, median (range)	74 (41–88)	76 (48–96)	0.529	57 (54–74)	64 (41–75)	0.141
Gender						
Male	11 (38%)	18 (62%)	0.712	3 (8%)	36 (92%)	0.424
Female	10 (33%)	20 (67%)		4 (15%)	22 (85%)	
Primary tumor location						
colon	10 (34.5%)	19 (65.5%)	0.861	5 (11%)	42 (89%)	1.000
rectum	11 (37%)	19 (63%)		2 (11%)	16 (89%)	
Clinical TNM						
cT1‐2	11 (52%)	10 (48%)	**0.045**			
cT3‐4	10 (26%)	28 (74%)				
cN‐	9 (45%)	11 (55%)	0.280			
cN+	12 (31%)	27 (69%)				
Pathological TNM						
pT1‐2	4 (50%)	4 (50%)	0.438			
pT3‐4	17 (37%)	34 (63%)				
pN‐	11 (38%)	18 (62%)	0.712			
pN+	10 (33%)	20 (67%)				
Number of metastatic sites						
1				4 (15%)	22 (85%)	0.424
> 1				3 (8%)	36 (92%)	
Unresected primary tumor						
Yes				5 (11%)	39 (89%)	1.000
No				2 (19%)	19 (90%)	

Bold values represent *P* < 0.05.

a Clinical and pathological TNM stages were not available for this cohort.

### 3.7 ctDNA dynamics in mCRC patients

**Fig. 3:**
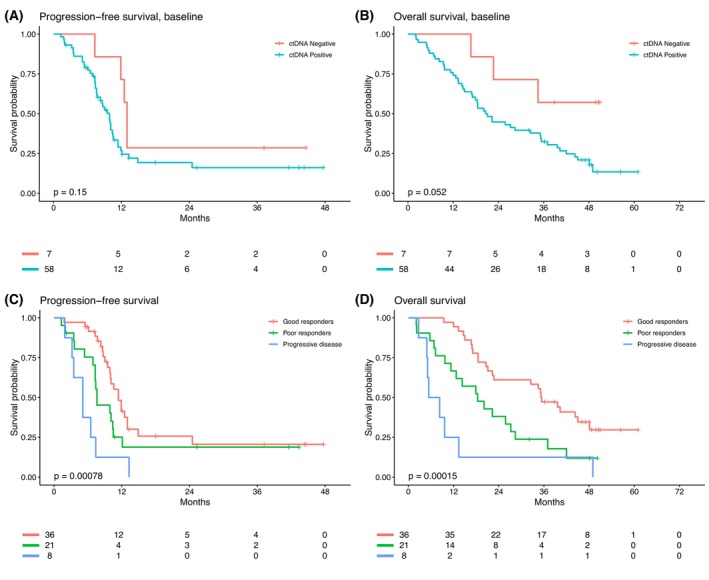
Kaplan–Meier plots showing survival curves of patients with metastatic colorectal cancer according to ctDNA measurements (*N* = 65). (A) Progression‐free survival (PFS) and (B) overall survival (OS) of patients with a ctDNA‐negative or ctDNA‐positive sample at baseline. (C) PFS and (D) OS of patients according to ctDNA positivity and fraction changes from baseline to after the first treatment cycle. Patients were classified as progressive disease if they presented an increase in ctDNA fraction; poor responders, if they were consistently ctDNA‐positive with ≤ 75% decrease, or stable ctDNA fraction after the first treatment cycle. Patients who were negative after treatment, regardless of changes in ctDNA fraction, and those consistently positive with a significant decrease of > 75% in ctDNA fraction were classified as good responders. Log rank was used to compare survival curves.

The median PFS of patients with a ctDNA‐negative sample at baseline was 13 months (95% CI 11.9—not reached (NR)) compared with 9.9 months (95% CI 7.7–10.6) in those with a ctDNA‐positive sample (log rank *P* = 0.15, HR = 1.94, 95% CI 0.76–4.95) (Fig. 3A). Median OS for patients with a ctDNA‐negative sample at baseline was not reached, while a median OS of 20.9 months (95% CI 17.1–35.2) was observed in ctDNA‐positive patients at baseline (log rank *P* = 0.05, HR = 3.03, 95% CI 0.94–9.78) (Fig. 3B).

When looking at changes from baseline to after the first cycle of treatment, we observed that 48 patients were ctDNA‐positive both at baseline and after treatment (positive–positive, PP), 10 were positive at baseline and became negative (positive–negative, PN), and seven were negative in both samples (negative–negative, NN) (Table S6). None of the patients with a ctDNA‐negative sample became positive after the first treatment cycle. No statistically significant differences in PFS or OS were observed when comparing these groups.

The variation in ctDNA fraction between the first and second samples was diverse among patients. While some patients experienced a significant increase (*N* = 8), others had stable values (*N* = 24), or a significant decrease was observed (*N* = 33). Based on ctDNA‐RECIST [14], patients were classified into progressive disease (PD, *N* = 8), partial response (PR, *N* = 28), stable disease (SD, *N* = 24), and near complete response (NCR, *N* = 5); no patients achieved a complete response.

Kaplan–Meier curves and survival analyses showed a significant difference in PFS (log rank *P* = 0.00013) and OS (log rank *P* = 0.0049) among the groups (Fig. S7A,B). A higher HR was observed for patients with PD (Table 4), while the distinction between PR, SD, and NCR was less clear. Further analyses demonstrated significant differences in PFS and OS based on ctDNA fraction decrease (> 75% or ≤ 75%) (Fig. S7C,D).

**Table 4:** Cox regression analysis across classification methods. CI, confidence interval; HR, hazard ratio; NR, not reached; OS, overall survival; PFS, progression‐free survival; RECIST, Response Evaluation Criteria in Solid Tumors.

Classification	*N*	PFS				OS			
		Median (95% CI)	HR	95% CI	*P*‐value	Median (95% CI)	HR	95% CI	*P*‐value
ctDNA‐RECIST									
Near complete response	5	NR (11.4‐NR)	Reference			39.6 (32.5‐NR)	Reference		
Stable disease	24	12.1 (10.1‐NR)	1.99	0.45–8.71	0.400	30.15 (20.1‐NR)	1.01	0.34–3.04	> 0.9
Partial response	28	8.7 (7.6–10.5)	4.78	1.11–20.6	**0.036**	21.7 (17.9–40.3)	1.34	0.46–3.91	0.600
Progressive disease	8	5.1 (3.5‐NR)	10.20	2.13–48.5	**0.004**	6.85 (5.1‐NR)	4.22	1.25–14.2	**0.020**
ctDNA decrease									
> 75%	22	10.1 (9.5‐NR)	Reference			37.5 (21.1‐NR)	Reference		
≤ 75%	11	7.4 (7.3‐NR)	4.42	1.81–10.8	**0.0005**	14.3 (11.3‐NR)	4.42	1.86–10.5	**< 0.001**
ctDNA combination group									
Good responders	36	11.4 (10.0–14.9)	Reference			35.3 (22.3‐NR)	Reference		
Poor responders	21	7.6 (7.3–12.1)	1.71	0.90–3.25	0.10	18.4 (11.3–37)	2.15	1.16–3.99	**0.015**
Progressive disease	8	5.1 (3.5‐NR)	4.36	1.91–9.92	**< 0.001**	6.85 (5.1‐NR)	4.73	2.09–10.7	**< 0.001**

Bold values represent *P* < 0.05.

To refine the classification, patients were combined into three categories, with increasing PFS and OS (Fig. 3C,D): Patients with (1) progressive disease, who presented an increase in ctDNA fraction; (2) poor responders, which correspond to patients consistently positive with ≤ 75% decrease, or stable ctDNA fraction after the first treatment cycle; and (3) good responders, patients who were negative after the treatment, regardless of changes in ctDNA fraction, or those consistently positive with a significant decrease of > 75% in ctDNA fraction. The median survival and Cox proportional hazard ratios for the different classification methods are detailed in Table 4.

## 4 Discussion

CRC remains a significant global health challenge due to its high incidence and mortality. ctDNA has emerged as a promising biomarker for real‐time, noninvasive monitoring that can support risk stratification, early relapse detection, and evaluation of treatment response—ultimately improving clinical outcomes. In this study, we developed and validated a multiplex MS‐ddPCR assay designed to detect ctDNA independently of tumor mutational profiling. By combining tumor‐specific and tissue‐conserved methylation markers, we aimed to create a broadly applicable, cost‐effective, and rapid test suitable for routine clinical use across clinical stages and independent of mutational status. The assay demonstrated high specificity (96.7%) and sensitivity for ctDNA detection in both localized (64.4%) and mCRC (89.2%), and importantly, dynamic changes in ctDNA levels were associated with treatment outcomes in metastatic cases.

The rationale for including tissue‐conserved methylation markers was supported by prior studies showing that cfDNA in healthy individuals primarily originates from white blood cells (55%), erythrocyte progenitors (30%), vascular endothelial cells (10%), and hepatocytes (1%) [11]. This work laid the foundation for using methylation signatures to trace the tissue origin of cfDNA, showing that tissue‐conserved DNA methylation markers coming from diseased organs can be detected in patient plasma samples [12]. Building on this, we previously developed and applied a three‐marker methylation panel (*NPY*, *KANK1*, and *GAL3ST3*) in the perioperative setting of colorectal liver metastases, demonstrating that pre‐ and postoperative negative ctDNA levels were strongly associated with prolonged PFS and OS. These findings supported its potential to guide the timing of liver intervention and inform postoperative treatment planning [36]. Advancements in ddPCR technology, including expanded fluorophore capabilities, enabled us to revise our multiplex assay to incorporate additional methylation targets to enhance both sensitivity and specificity. We confirmed that the selected methylated biomarkers exhibited higher levels in genomic DNA from colorectal tissue than from blood‐derived DNA (Fig. 1). As expected, the tissue‐conserved markers *FRMD4B*, *GDAP1L1*, and *CELF2* showed consistently higher methylation in normal colorectal tissue compared with blood (Fig. S4). Expanding the number of markers improved not only the assay's sensitivity but also its specificity in correctly identifying CRC patients. This improvement was particularly evident in plasma samples from patients with localized disease, where the sensitivity of individual markers ranged from 42.4% to 66.1%, in contrast to 83.1% to 90.8% in samples from patients with mCRC (Table S5). Although *NPY* and *GDAP1L1* presented high sensitivity in patients with localized (66.1%) and metastatic (90.8%) disease, respectively, their specificities were rather low (*NPY* = 73.3% and *GDAP1L1* = 78.3%). Ultimately, the best performance in terms of accuracy was achieved when using the full MS‐ddPCR multiplex, particularly for detecting ctDNA in patients with localized disease (Table S5).

Although DNA methylation is generally considered a stable epigenetic feature, some loci may be influenced by clinical variables, such as age [37]. In our analysis of patients with localized disease, methylated *NPY* and *FRMD4B* levels were significantly higher in individuals with cT3–4 or cN+ tumors, suggesting an association with tumor burden. A similar trend was observed for *GDAP1L1*, although statistical significance was reached only for the cT comparison. Notably, cT stage was also significantly associated with the proportion of ctDNA‐positive cases, with 74% of cT3–4 cases testing positive. These findings are in line with previous studies reporting higher ctDNA levels and detection rates in more advanced disease stages [7, 22, 38, 39]. While we observed an increasing trend in ctDNA positivity with advancing clinical stages, the differences were not statistically significant, likely due to the limited number of patients in each category. No additional associations were found between individual marker levels and clinical or histopathological features. Several studies employing different methodologies have reported ctDNA detection in plasma from CRC patients with varied sensitivity and specificity [4, 7, 13, 19, 25, 39]. Our primary objective in developing this MS‐ddPCR multiplex assay was to create a sensitive, broadly accessible test that could be implemented locally, without reliance on centralized laboratories or proprietary sequencing platforms. We aimed to minimize costs and turnaround times, which are often higher in tumor‐informed workflows. While quantitative PCR (qPCR) offers a faster and lower‐cost alternative, ddPCR provides superior sensitivity, reproducibility, and absolute quantification—critical for reliable assessment of serial samples. Although the assay was not designed for early detection, its robust performance encourages further investigation into its applicability in premalignant lesions and early‐stage colorectal cancer.

In the context of mCRC, ctDNA has emerged as a promising biomarker for prognostication and early assessment of treatment response [8, 9, 17, 20, 23]. In our cohort, baseline ctDNA positivity was not significantly associated with PFS or OS. However, dynamic changes in ctDNA levels from baseline to after the first treatment cycle were significantly correlated with both outcomes (Fig. 3, Table 4), supporting the value of ctDNA kinetics as a more informative metric than static measurements. This aligns with growing evidence indicating that ctDNA dynamics may enable earlier and more accurate assessment of treatment efficacy in mCRC.

A ctDNA‐based response classification, termed ctDNA‐RECIST, has recently been proposed using ddPCR‐derived confidence intervals to define significant changes in ctDNA levels between consecutive time points [40]. A change is considered significant when the 95% CI of two measurements does not overlap. This classification mirrors conventional RECIST criteria but includes an additional ‘near complete response’ category, which is uniquely measurable by ctDNA. In our cohort, applying ctDNA‐RECIST allowed for clear stratification of patients, with pronounced differences in PFS and OS between those with progressive disease and those showing near complete response (Fig. S7A,B, Table 4). However, there was less distinction between patients classified as having stable or partial response, suggesting that further refinement of these categories may be beneficial.

We also observed that patients with a > 75% reduction in ctDNA after one treatment cycle had significantly improved PFS and OS (Fig. S7C,D, Table 4), consistent with previous findings [8, 9, 20, 38, 41, 42]. However, the optimal threshold for ctDNA change that best predicts clinical outcomes remains variable across studies. In the PLACOL study, a deep early decrease of > 80% in ctDNA concentration was significantly associated with favorable therapeutic responses, highlighting its prognostic value in evaluating first‐ or second‐line chemotherapy efficacy in 63 mCRC patients [41]. Similarly, Tie *et al*. [43] reported that a ≥ 10‐fold reduction in ctDNA after the first chemotherapy cycle was associated with improved PFS in 44 mCRC patients. Using the median ctDNA change between baseline and before the third cycle of treatment (1 month after treatment start), Taïeb *et al*. identified significant differences in PFS and OS in 99 patients with dMMR/MSI‐H mCRC who were treated with an immune checkpoint inhibitor (avelumab) or standard chemotherapy. Multivariable analysis showed an association of lack of ctDNA response with an increased risk of disease progression and death in the avelumab group (HR, 7.27; 95% CI, 2.23–23.7; *P* = 0.001) but not in the chemotherapy group (HR, 1.61; 95% CI, 0.66–3.93; *P* = 0.30) [44]. Although the limited sample sizes in each study preclude a precise definition of a universal cutoff, the consistent findings across studies underscore the strong association between early ctDNA dynamics and treatment efficacy. These data support the growing consensus that ctDNA kinetics represent a robust biomarker for predicting outcomes and guiding personalized treatment strategies in mCRC. In addition to early treatment assessment, longitudinal monitoring of ctDNA could further enhance clinical decision‐making by enabling timely detection of disease progression or resistance, potentially prompting earlier therapeutic adjustments.

While the results of this study are promising and demonstrate the potential clinical utility of the MS‐ddPCR assay, some limitations should be considered. First, tumor stage information was not available for the analyzed tissue samples, limiting our ability to correlate methylation marker expression with early disease stages. Second, although bisulfite conversion is essential for methylation analysis, it can result in cfDNA loss, which may affect assay sensitivity. The relatively limited number of cases also restricts statistical power, and the absence of a validation cohort precludes external confirmation of the findings. Future studies should aim to validate the assay in larger and more diverse patient populations and across different clinical settings.

## 5 Conclusions

In conclusion, we have identified tissue‐conserved methylated markers, which, combined with known tumor‐specific markers, can detect ctDNA with high accuracy in patients with localized and mCRC. It can be used as a universal liquid biopsy test that is independent of tumor profiling, at a relatively low cost and fast turnaround time. The resulting MS‐ddPCR multiplex assay detection of ctDNA kinetics provides an early and powerful assessment of treatment response and prognosis, holding potential to guide personalized therapeutic strategies. It also has the advantage of facilitating follow‐up in mCRC patients with unknown mutational status.

## Conflict of interest

The authors declare no conflict of interest.

## Author contributions

LMC, RFA, LHJ, and TFH contributed to the conceptualization. RFA and TFH contributed to the experimental design. LR, JL, MMA, CT, JK, LMC, and RFA contributed to the data collection. MMA, LMC, and RFA contributed to the data analysis. LMC and RFA contributed to the writing and editing. All authors reviewed and approved the final manuscript version.

## Supporting information


**Fig. S1.** Correlation between results obtained with the original, previously published, *NPY* assay and the new assay described in this study (*N* = 65).

**Fig. S2.** Amplitude fluorescence plots in (A) 1D and (B) 2D of a 6‐target methylation‐specific multiplex droplet digital PCR assay analysis of Universal Methylated Human DNA Standard (Zymo Research) using the QX Manager Software v.2.2.

**Fig. S3.** Correlation between expected and measured copies per reaction of a 6‐point 2‐fold dilution series of the Universal Methylated Human DNA Standard (Zymo Research) in a constant background of 5600 copies of BSC genomic DNA for each marker in duplex or multiplex assays.

**Fig. S4.** Methylation levels of colorectal cancer‐specific (*NPY* and *CLIP4*) and tissue‐conserved (*FRMD4B*, *GDAP1L1*, and *CELF2*) markers in normal tissue samples from colon (*N* = 10) and rectum (*N* = 10).

**Fig. S5.** Association of methylation levels of each marker and clinical and histopathological characteristics of colorectal cancer patients diagnosed with localized disease (*N* = 59).

**Fig. S6.** Association of methylation levels of each marker and clinical characteristics of metastatic colorectal cancer patients (*N* = 65).

**Fig. S7.** Kaplan Meier plots showing survival curves of patients with metastatic colorectal cancer according to changes in ctDNA measurements from baseline to after the first treatment cycle.

**Table S1.** Datasets used for identifying colon‐specific methylation sites.
**Table S2.** Description of the oligos used in the multiplex droplet digital PCR.
**Table S3.** ctDNA‐RECIST criteria modified from Spindler KG & Jakobsen A (2023).
**Table S4.** Classification of changes in biomarker fraction from baseline to after the first treatment cycle detected in metastatic colorectal cancer patients' plasma samples.
**Table S5.** Sensitivity, specificity, positive predictive value (PPV), negative predictive value (NPV), and accuracy of each marker and the combined methylation‐specific droplet digital PCR (MS‐ddPCR) multiplex assay.
**Table S6.** Description of markers detected above the limit of blank (+) and samples classified as ctDNA‐positive (+) for every patient with metastatic colorectal cancer.

## Data Availability

The data that support the findings of this study are available from the corresponding author [rikke.fredslund.andersen@rsyd.dk] upon reasonable request.
