# Supplementary figures and images for: Detection of circulating tumor DNA in colorectal cancer patients using a methylation‐specific droplet digital PCR multiplex
Source: Mol Oncol. 2025 Nov 14;20(4):904–19. doi: 10.1002/1878-0261.70161 (PMC13060638; doi:10.1002/1878-0261.70161)

**A** *ALB copies/ml*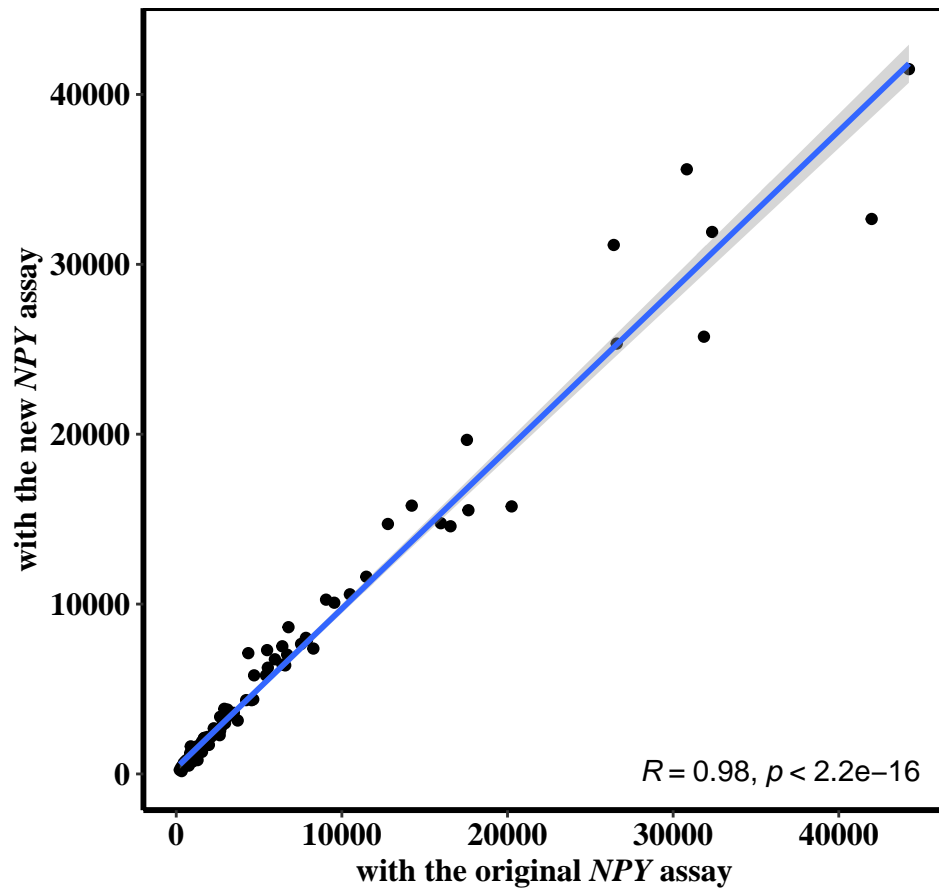**B** *NPY copies/ml*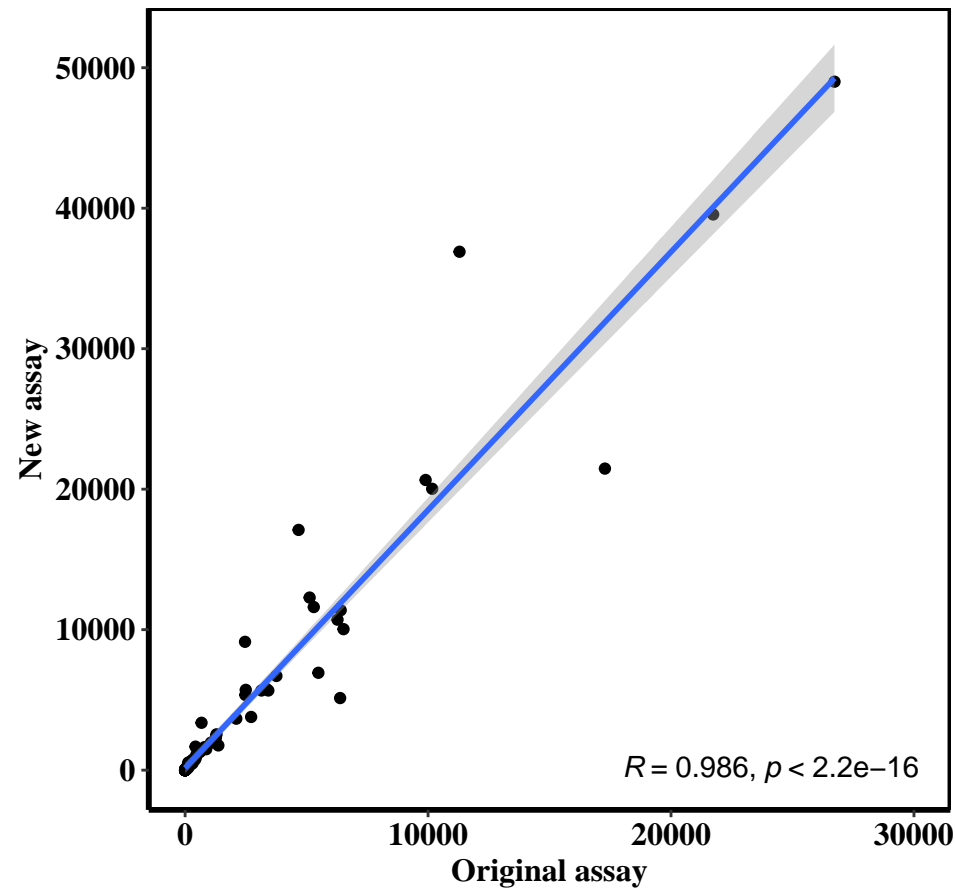**C** % NPY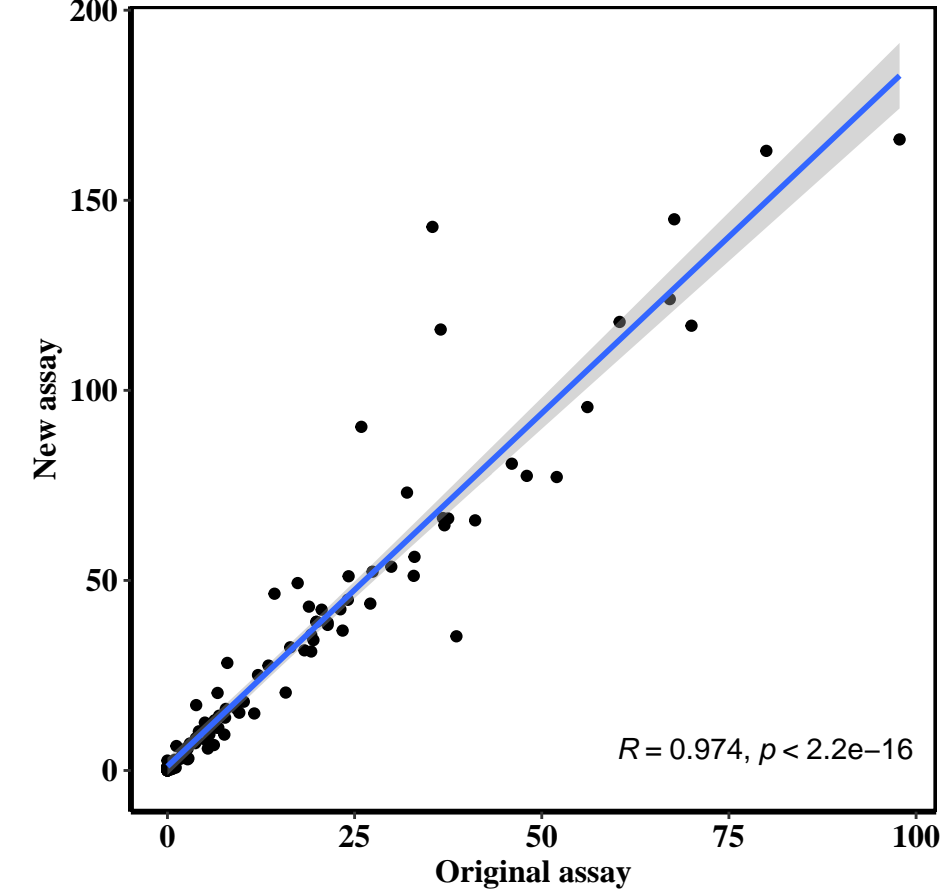

Supplement: Supplementary file 1 — Fig. S1. Correlation between results obtained with the original, previously published, NPY assay and the new assay described in this study (N = 65). [file MOL2-20-904-s008.pdf]

**A**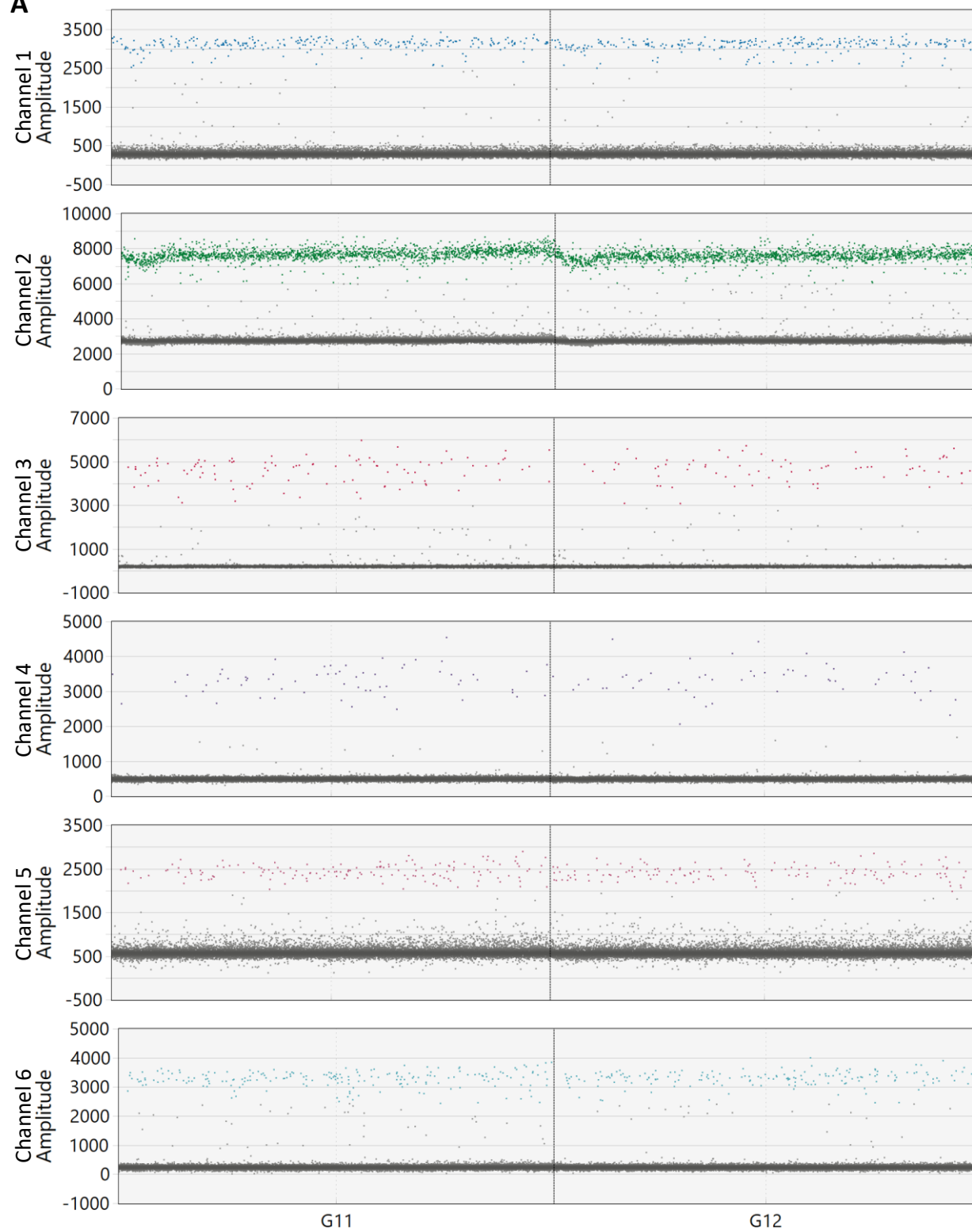**B**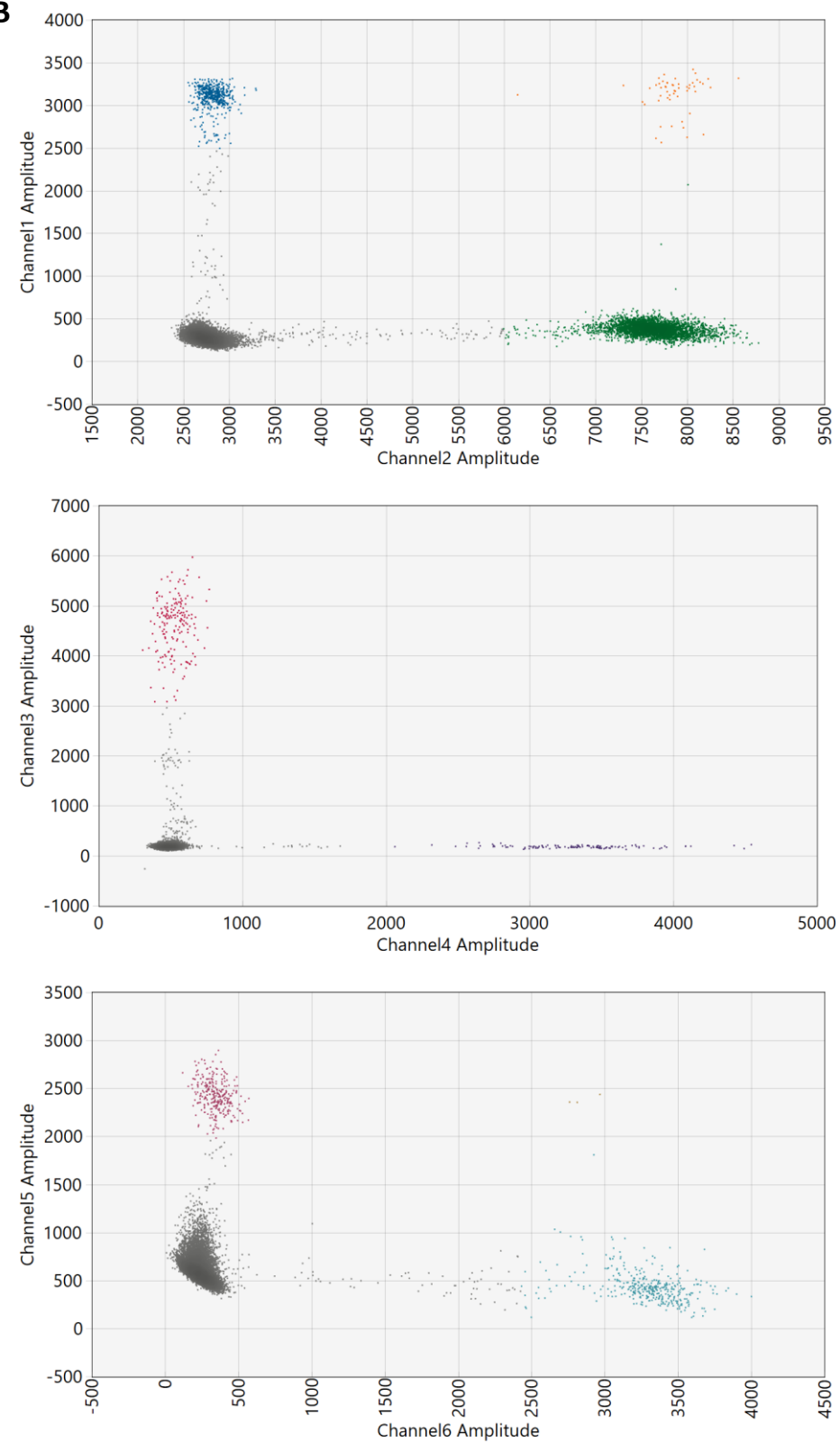

Supplement: Supplementary file 2 — Fig. S2. Amplitude fluorescence plots in (A) 1D and (B) 2D of a 6‐target methylation‐specific multiplex droplet digital PCR assay analysis of Universal Methylated Human DNA Standard (Zymo Research) using the QX Manager Software v.2.2. [file MOL2-20-904-s005.pdf]

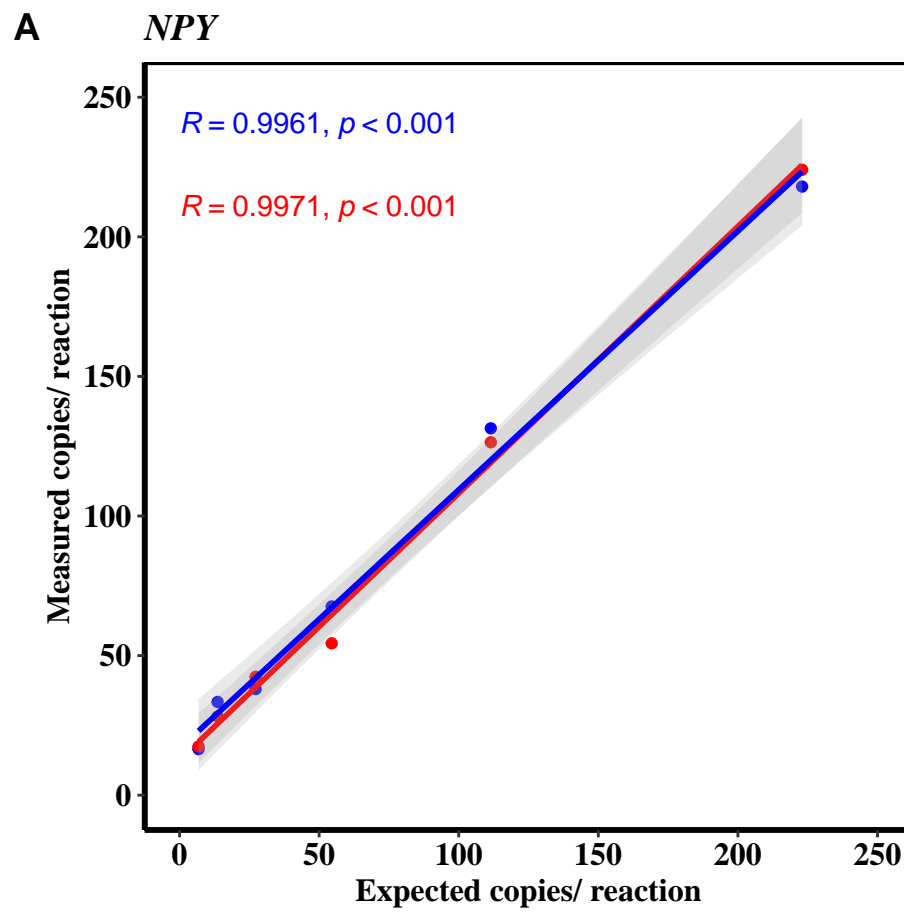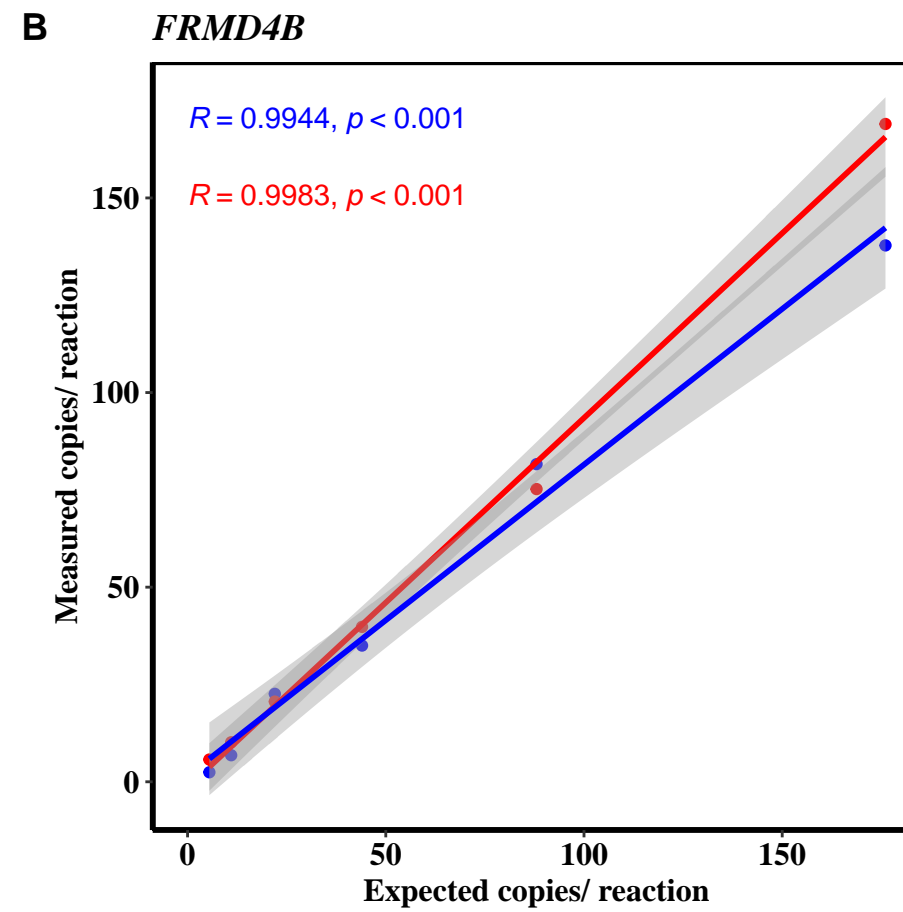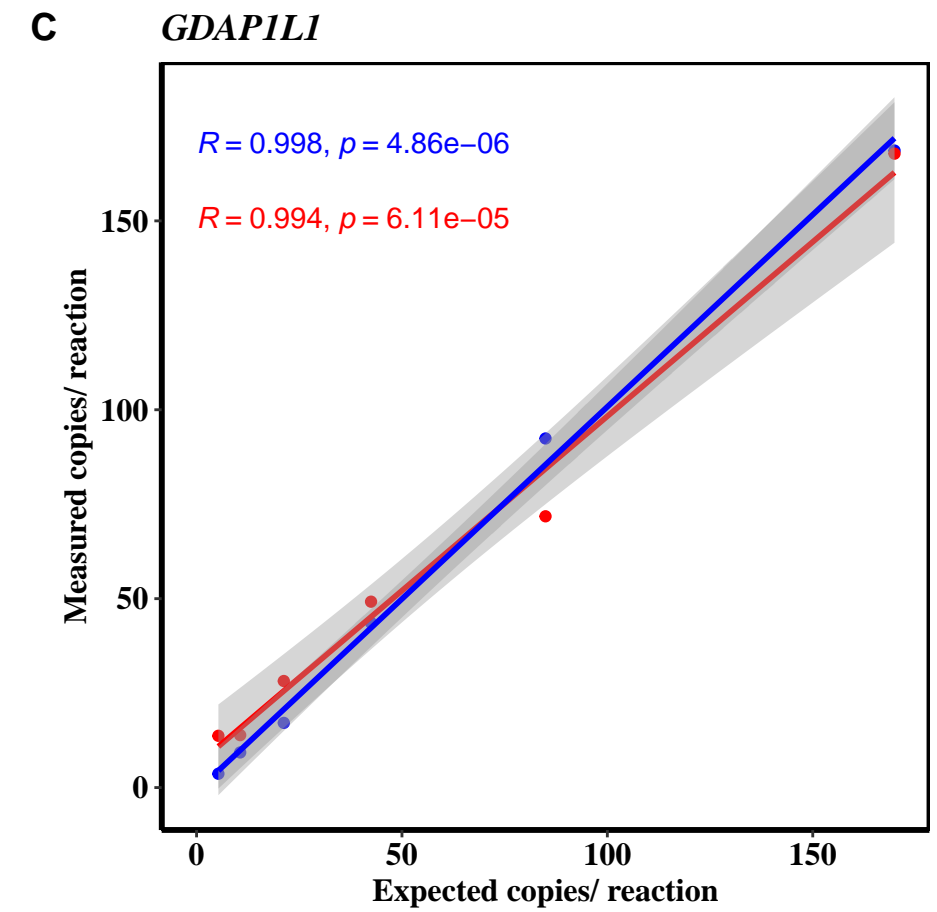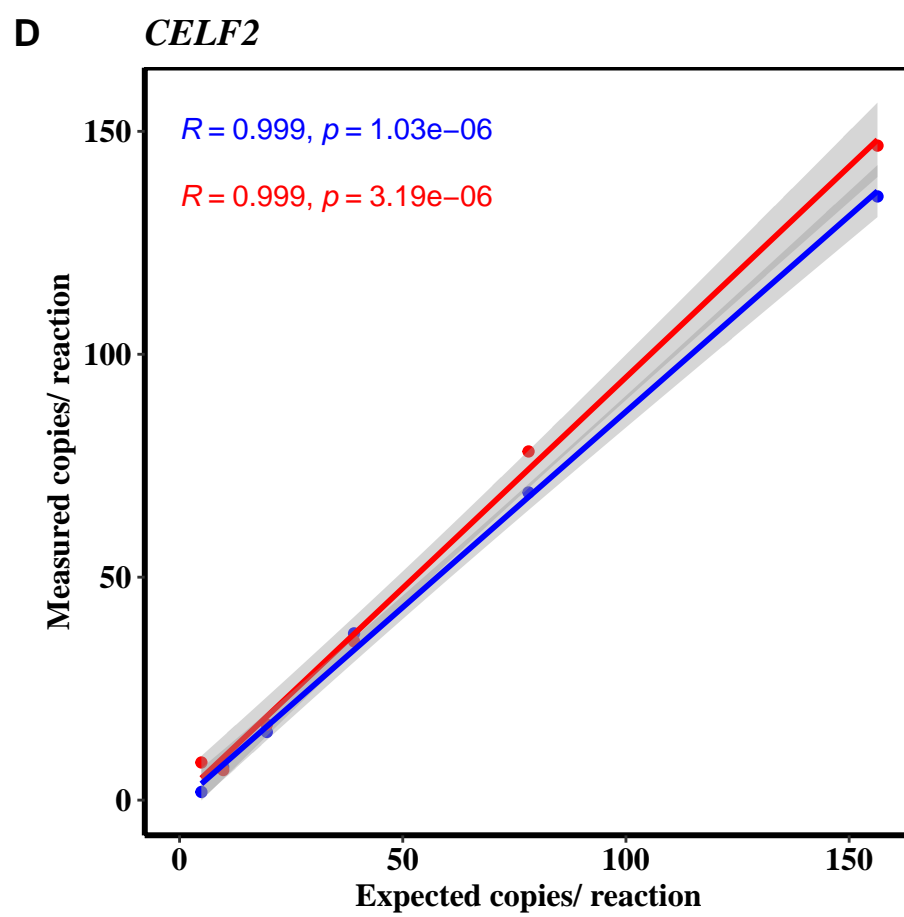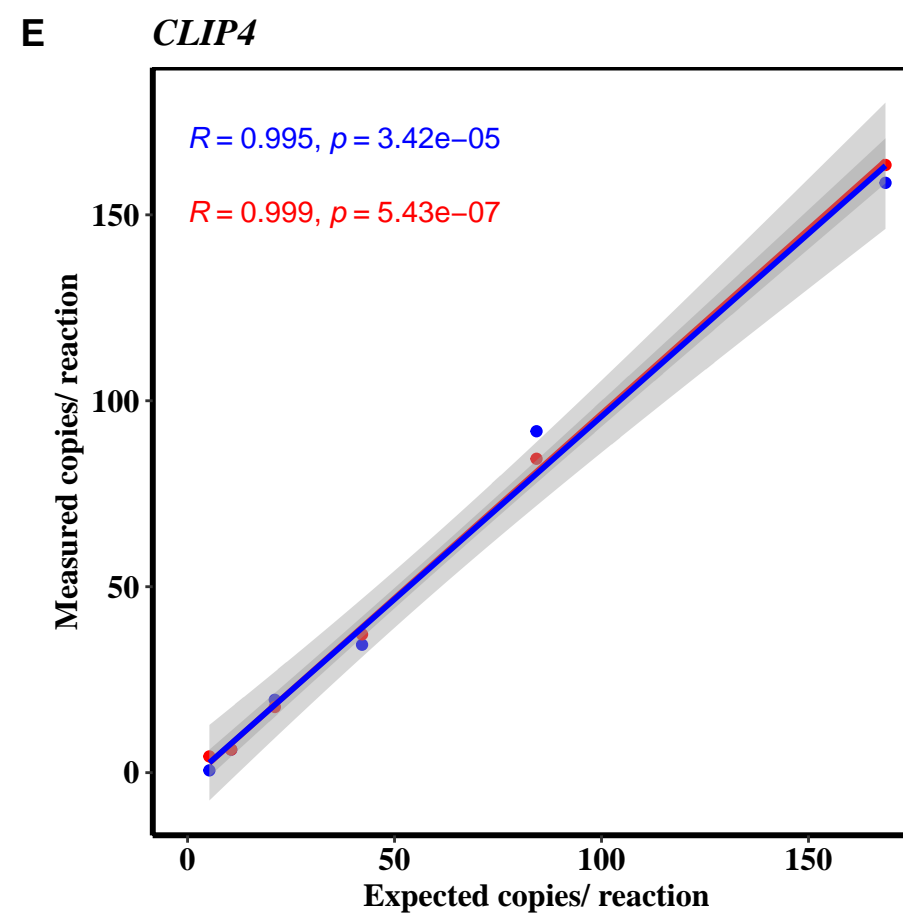

Duplex Multiplex

Supplement: Supplementary file 3 — Fig. S3. Correlation between expected and measured copies per reaction of a 6‐point 2‐fold dilution series of the Universal Methylated Human DNA Standard (Zymo Research) in a constant background of 5600 copies of BSC genomic DNA for each marker in duplex or multiplex assays. [file MOL2-20-904-s007.pdf]

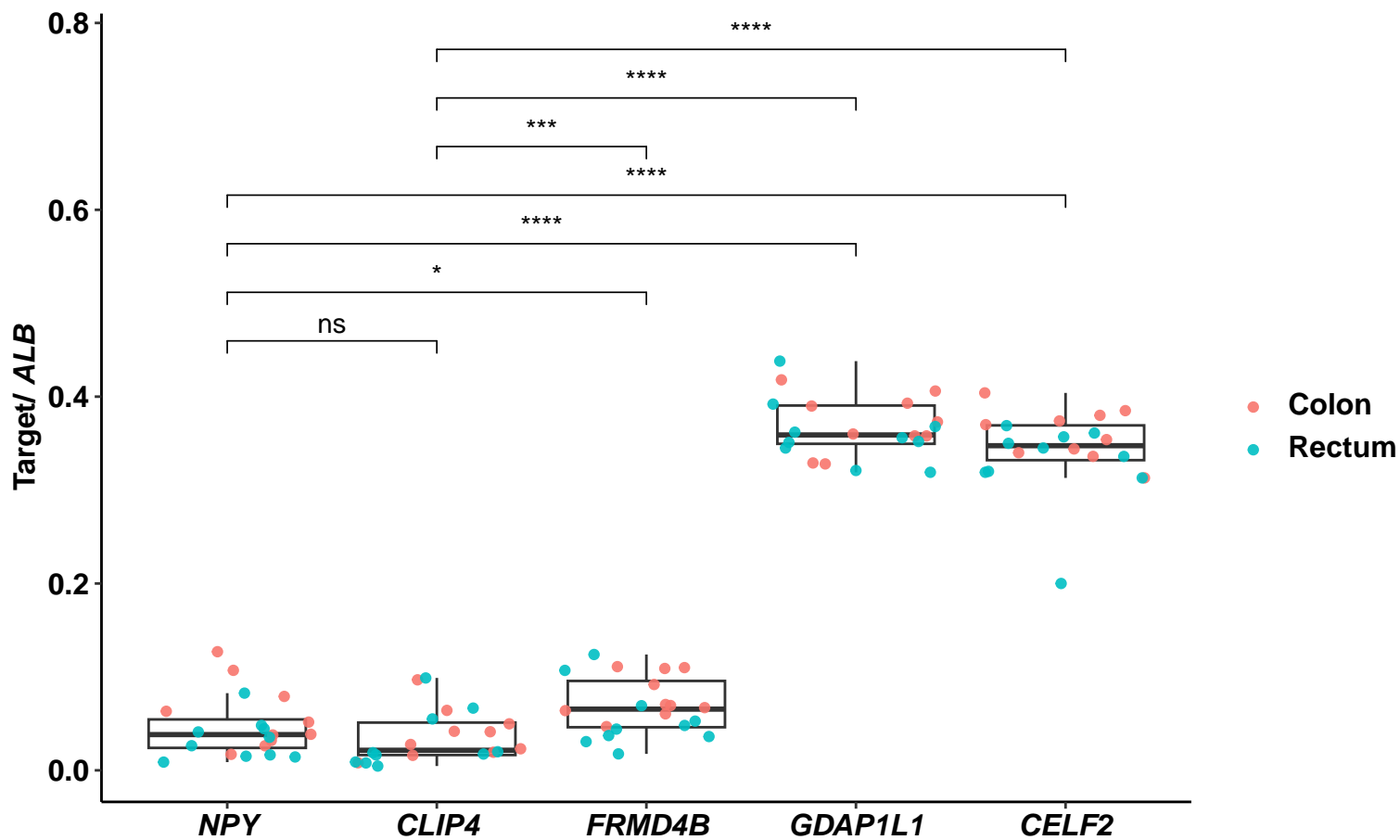

Supplement: Supplementary file 4 — Fig. S4. Methylation levels of colorectal cancer‐specific (NPY and CLIP4) and tissue‐conserved (FRMD4B, GDAP1L1, and CELF2) markers in normal tissue samples from colon (N = 10) and rectum (N = 10). [file MOL2-20-904-s002.pdf]

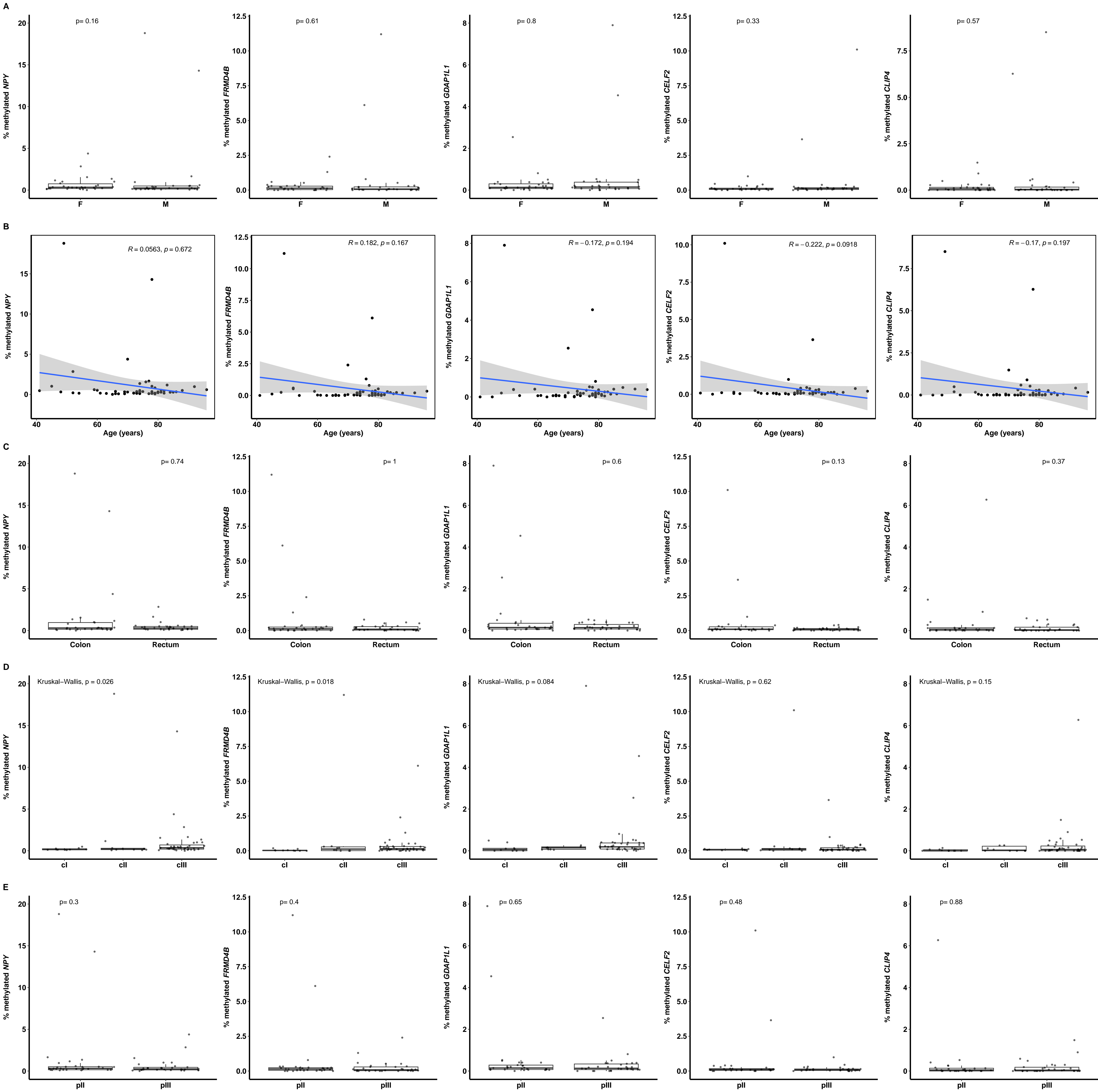

Supplement: Supplementary file 5 — Fig. S5. Association of methylation levels of each marker and clinical and histopathological characteristics of colorectal cancer patients diagnosed with localized disease (N = 59). [file MOL2-20-904-s003.pdf]

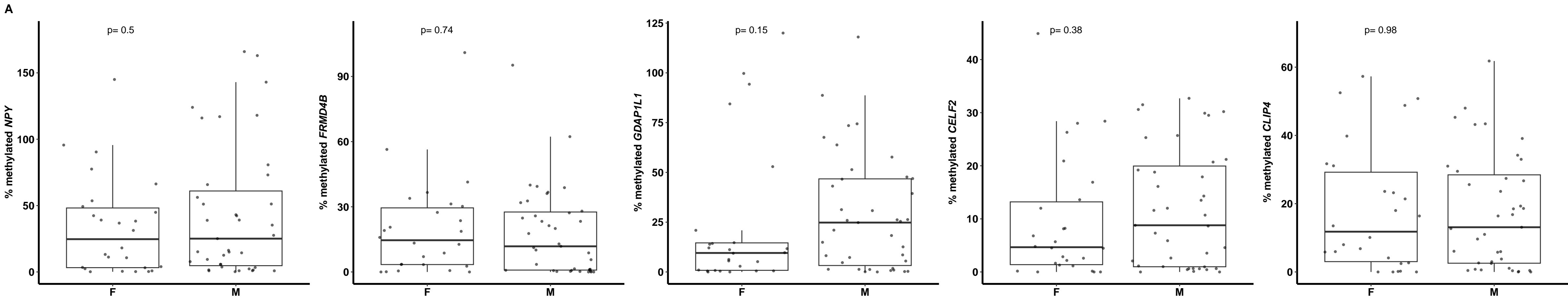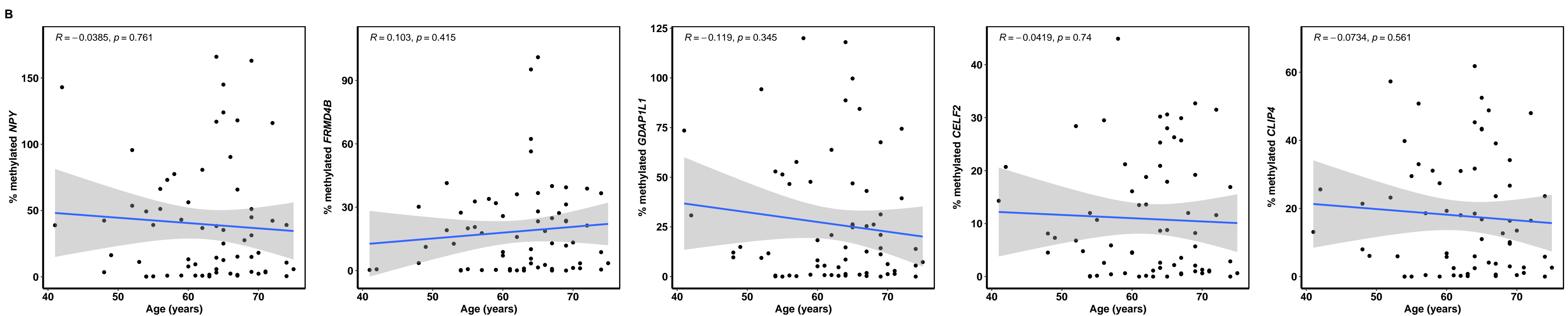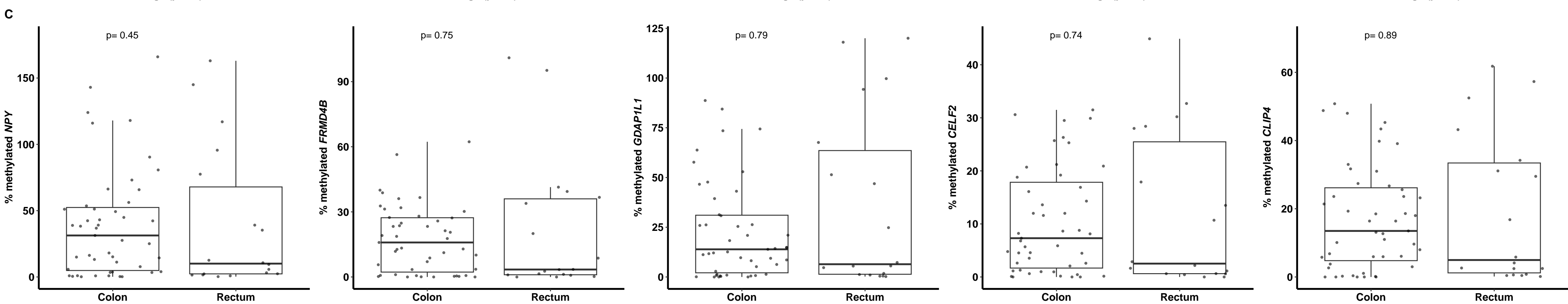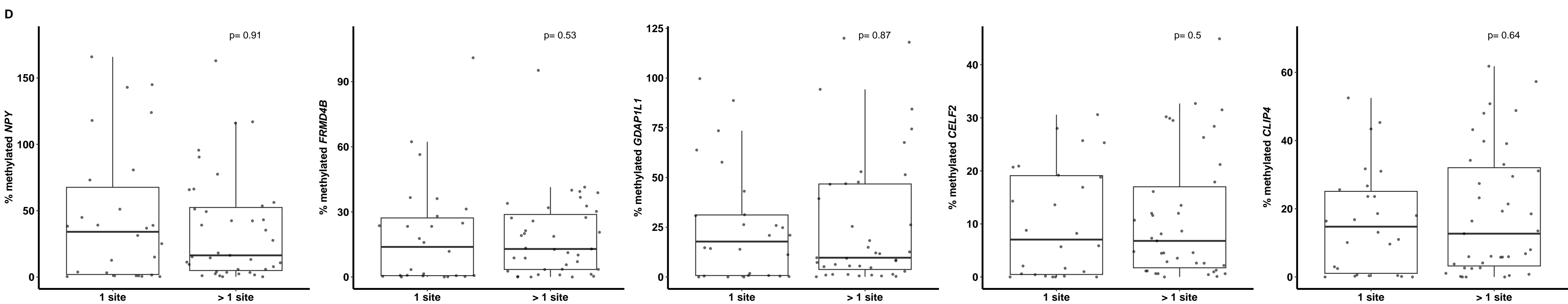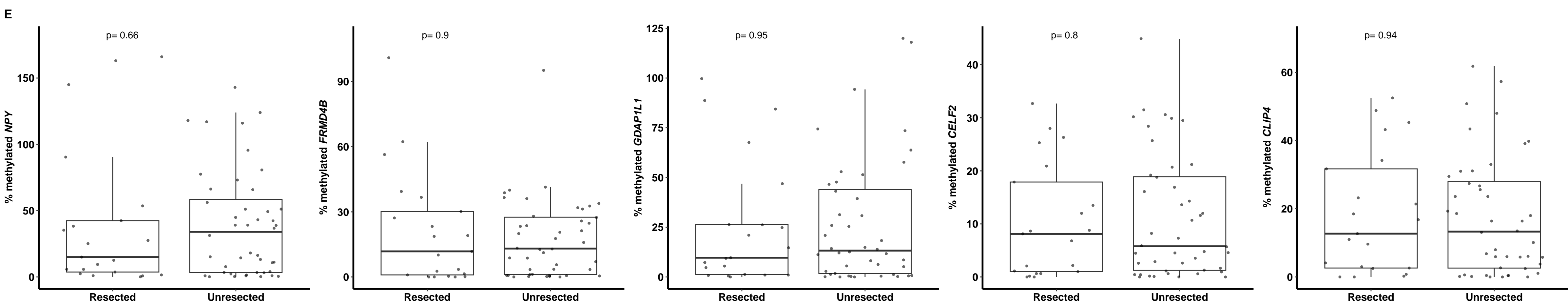

Supplement: Supplementary file 6 — Fig. S6. Association of methylation levels of each marker and clinical characteristics of metastatic colorectal cancer patients (N = 65). [file MOL2-20-904-s001.pdf]
