# Supplementary material for: Detection of circulating tumor DNA in colorectal cancer patients using a methylation‐specific droplet digital PCR multiplex
Source: Mol Oncol. 2025 Nov 14;20(4):904–19. doi: 10.1002/1878-0261.70161 (PMC13060638; doi:10.1002/1878-0261.70161)
Supplement: Supplementary file 7 — Fig. S7. Kaplan Meier plots showing survival curves of patients with metastatic colorectal cancer according to changes in ctDNA measurements from baseline to after the first treatment cycle. [file MOL2-20-904-s004.pdf]

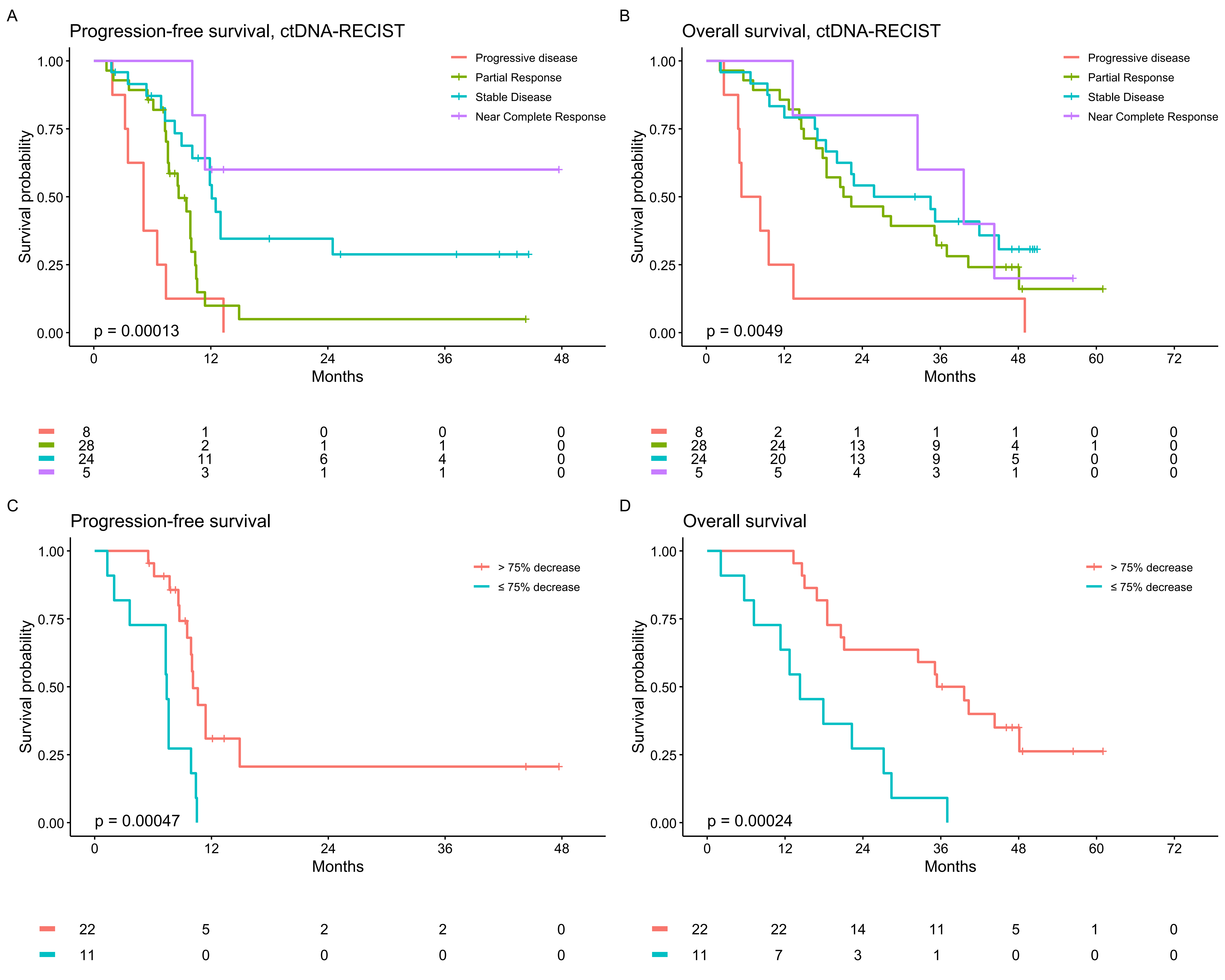
A
Progression-free survival, ctDNA-RECIST
Progressive disease
Partial Response
Stable Disease
Near Complete Response
Survival probability
1.00
0.75
0.50
0.25
0.00
p = 0.00013
0 12 24 36 48
Months
8 1 0 0 0
28 2 1 1 0
24 11 6 4 0
5 3 1 1 0
B
Overall survival, ctDNA-RECIST
Progressive disease
Partial Response
Stable Disease
Near Complete Response
Survival probability
1.00
0.75
0.50
0.25
0.00
p = 0.0049
0 12 24 36 48 60 72
Months
8 2 1 1 1 0 0
28 24 13 9 4 1 0
24 20 13 9 5 0 0
5 5 4 3 1 0 0
C
Progression-free survival
> 75% decrease
≤ 75% decrease
Survival probability
1.00
0.75
0.50
0.25
0.00
p = 0.00047
0 12 24 36 48
Months
22 5 2 2 0
11 0 0 0 0
D
Overall survival
> 75% decrease
≤ 75% decrease
Survival probability
1.00
0.75
0.50
0.25
0.00
p = 0.00024
0 12 24 36 48 60 72
Months
22 22 14 11 5 1 0
11 7 3 1 0 0 0
